# Differential effects of exposure to low-light or high-light open-field on anxiety-related behaviors; relationship to c-Fos expression in serotonergic and non-serotonergic neurons in the dorsal raphe nucleus

**DOI:** 10.1016/j.brainresbull.2006.12.009

**Published:** 2007-01-16

**Authors:** J. Adriaan Bouwknecht, Francesca Spiga, Daniel R. Staub, Matthew W. Hale, Anantha Shekhar, Christopher A. Lowry

**Affiliations:** 1 Henry Wellcome Laboratories for Integrative Neuroscience and Endocrinology, University of Bristol, Whitson Street, Bristol, BS1 3NY, United Kingdom; 3 Department of Psychiatry, Pharmacology and Toxicology, Indiana University School of Medicine, Indianapolis, IN 46202, USA

**Keywords:** 5-hydroxytryptamine, anxiety, c-Fos, immediate-early gene, serotonin, tryptophan hydroxylase

## Abstract

Serotonergic systems arising from the mid-rostrocaudal and caudal dorsal raphe nucleus (DR) have been implicated in the facilitation of anxiety-related behavioral responses by anxiogenic drugs or aversive stimuli. In this study we attempted to determine a threshold to engage serotonergic neurons in the DR following exposure to aversive conditions in an anxiety-related behavioral test. We manipulated the intensity of anxiogenic stimuli in studies of male Wistar rats by leaving them undisturbed (CO), briefly handling them (HA), or exposing them to an open-field arena for 15-min under low-light (LL: 8-13 lux) or high-light (HL: 400-500 lux) conditions. Rats exposed to HL conditions responded with reduced locomotor activity, reduced time spent exploring the center of the arena, a lower frequency of rearing and grooming, and an increased frequency of facing the corner of the arena compared to LL rats. Rats exposed to HL conditions had small but significant increases in c-Fos expression within serotonergic neurons in subdivisions of the rostral DR. Exposure to HL conditions did not alter c-Fos responses in serotonergic neurons in any other DR subdivision. In contrast, rats exposed to the open-field arena had increased c-Fos expression in non-serotonergic cells throughout the DR compared to CO rats, and this effect was particularly apparent in the dorsolateral part of the DR. We conclude that exposure to HL conditions, compared to LL conditions, increased anxiety-related behavioral responses in an open-field arena but this stimulus was at or below the threshold required to increase c-Fos expression in serotonergic neurons.

## 1. Introduction

Serotonin (5-hydroxytryptamine; 5-HT) influences a variety of behavioral and physiological processes including arousal, sleep-wake cycles, food intake, and anxiety-related behavior [15;21;31;36]. The central 5-HT network includes various nuclei in the brainstem of which the dorsal raphe nucleus (DR) is an important structure containing over 50% of all 5-HT neurons projecting to the forebrain [65]. Recently, it has become evident that the DR is a heterogeneous nucleus containing various anatomically, neurochemically, and functionally distinct subdivisions that have their own afferents and efferents and are responsive to different types of stimuli [16;30;31;37;43]. For example, serotonergic neurons within the rostral part of the DR give rise to serotonergic projections to forebrain systems involved in somatic motor responses such as the caudate putamen while serotonergic neurons in the mid-rostrocaudal and caudal DR give rise to serotonergic projections to other forebrain structures, such as the amygdala, involved in anxiety-related behavioral responses [5;15;39;59;65]. Therefore, it becomes important to use a high degree of topographical resolution when studying the role of serotonergic systems in anxiety- or stress-related responses [38]. The immediate-early gene product c-Fos is broadly expressed throughout the brain in response to a variety of challenges, which makes it a powerful tool to study intracellular responses of specific neurons in the brain under various conditions [10], particularly as it can be combined with characterization of the neurochemical properties of the responsive neurons.

A wealth of evidence supports an association between the neuronal activity of brainstem serotonergic neurons and the level of somatic motor activity or behavioral arousal. Single unit recording studies in behaving cats [47;55] and rats [71] indicate that the firing rates of the majority of serotonergic neurons in the DR are highly correlated with the level of behavioral arousal or sleep-wake states. These findings are supported by *in vivo* microdialysis studies demonstrating that increases in motor activity or behavioral arousal are associated with increases in extracellular concentrations of serotonin in multiple forebrain regions [53]. However, single unit recording studies in behaving cats have also identified subsets of serotonergic neurons that display patterns of neuronal activity that are not highly correlated with behavioral state [47;55], raising the possibility that subsets of serotonergic neurons may have different functional correlates.

Recent studies have revealed that serotonergic neurons within the mid-rostrocaudal and caudal regions of the DR may play a particularly important role in the facilitation of anxiety-related physiological or behavioral responses by anxiogenic drugs or uncontrollable, aversive stimuli. These parts of the DR have efferent projections to the amygdala (mid-rostrocaudal DR), as well as to the hippocampus and locus coeruleus (caudal DR) [28;29], components of a distributed neural system implicated in the modulation of anxiety- and fear-related behavioral responses [17]. Exposure to uncontrollable stress [16], or other anxiety-related stimuli including anxiogenic drugs such as the adenosine receptor antagonist caffeine, the serotonin 5-HT_2A/2C_ receptor agonist m-chlorophenyl piperazine (mCPP), and the partial inverse agonist at the benzodiazepine site on the GABA_A_ receptor N-methyl-beta-carboline-3-carboxamide (FG-7142) [1], the anxiety-related neuropeptide urocortin 2 [64], and social defeat [13] increase c-Fos expression within the midrostrocaudal and/or caudal parts of the DR, but not the rostral DR. However, the threshold for the selective activation of serotonergic systems within the mid-rostrocaudal and caudal DR has not been studied. Thus, it is unclear if serotonergic neurons in the mid-rostrocaudal and caudal regions of the DR also respond to mild anxiogenic stimuli and if the response is dependent on the aversiveness of the testing conditions. Here, we attempt to determine the stimulus threshold to induce c-Fos responses in the mid-rostrocaudal and caudal DR using a relatively mild unconditioned anxiety paradigm and to study the interaction with anxiety-like and locomotor behavioral responses.

The open-field exposure paradigm is a generally accepted animal model for measurement of anxiety-related behavior [2;45]. This paradigm is based on a conflict between the internal drive to explore a novel environment (based on the potential for rewarding outcomes) versus the internal drive to avoid a novel environment (based on the potential for aversive outcomes). Behavior in the open-field test is influenced by various factors, including genetic variability [56;68;69], gender [46], early life [6] and recent experiences of the individual [4], and the level of illumination of the open-field arena. Bright light can be used as an aversive stimulus in the open-field paradigm leading to an increase in anxiety-related behaviors [7;22;67]. Rodents are nocturnal and tend to avoid brightly-lit places. In addition, rodents also dislike wide-open spaces and prefer to stay close to vertical references such as walls, an innate behavioral response referred to as thigmotaxis [66].

In the present study we attempted to manipulate the aversiveness of the test conditions by comparing undisturbed control and gently-handled Wistar rats with rats that were exposed to low(8-13 lux) or high-light (400-500 lux) illumination in the open-field arena. The light intensities used in this study were comparable to previous behavioral studies using a similar behavioral test arena for an alternative anxiety paradigm, i.e. the social interaction test [14;54]. We predicted that serotonergic neurons within the mid-rostrocaudal and caudal DR would respond with greater increases in c-Fos expression in rats exposed to high-light illumination compared to rats exposed to low-light illumination in the open-field arena.

## 2. Materials and Methods

### Animals

A total of 32 male outbred Wistar rats (B & K Universal Ltd, Hull, UK) were used in this study. Rats and experimental procedures used in this study were identical to those used in a previous publication examining the effects of open-field exposure on c-Fos expression within the basolateral complex of the amygdala [20]; thus the brainstem sections in the present study were derived from the same brains as the forebrain sections processed in the previous study [20]. Briefly, rats arrived from the vendor weighing 233 ± 0.9 g and were housed socially at 23 °C for 4 days (4 rats per cage; RC1, 56 × 38 × 18 cm, North Kent Plastic Cages Ltd. Erith, UK) with *ad libitum* access to tap water and standard rat chow (CRM, B&K Universal Ltd., Hull, UK). Ten days prior to the first experimental day rats were single housed in RB3 cages (45 × 28 × 20 cm, North Kent Plastic Cages Ltd.). Throughout the experiment, rats were maintained on a 12L:12D light cycle, with lights on at 05.00 h; during the light phase, light in the home cages was below 100 lux. The average body weight on the test day had increased to 277 ± 2.3 g.

All animal procedures were approved by the University of Bristol Ethical Review Group and were conducted in accordance with Home Office guidelines and the UK Animals (Scientific Procedures) Act, 1986. In addition, all studies were consistent with the *NIH Guide for the Care and Use of Laboratory Animals* (NIH Publication No. 85-23) and were covered by Animal Welfare Assurance #A5057-01.

### Experimental design

Prior to the test day, all rats were weighed and handled daily for 2 min on 5 consecutive days to familiarize rats with the general procedures involved and to increase the stability of behavioral responses [25]. Each subject was randomly assigned to one of 4 treatment groups: i.e. control (CO), handled (HA), low-light open-field test (LL: 8-13 lux throughout the box) or high-light open-field test (HL: 400-500 lux throughout the box). CO rats were left undisturbed on the test day. For the HA group, the cage was moved from the holding room to the adjacent test room, the rat was picked up from the cage, put back immediately and returned to the holding room; this entire procedure took less than 45 sec. Rats exposed to LL and HL conditions in the open-field test were also moved to the adjacent room but were put in the open-field box for 15 min with either low- or high-light exposure, after which they were placed back in their home cage and were returned to the holding room. Rats were tested in pairs consisting of one CO or HA rat and one LL or HL rat (i.e. one rat remained in the holding room, while the other rat was tested in the open-field box). Two hours after the start of the test, the pair of rats was injected with an overdose of sodium pentobarbital (0.5-1.0 ml of Lethobarb (200 mg/ml), Fort Dodge, Southampton, UK) and brains were collected for immunohistochemistry. The selection of the 2 h time point was based on previous studies in which injections of anxiogenic drugs [1;61;62], or exposure to the elevated plus-maze [60] increased c-Fos expression 2 h later within anxiety-related neural circuits, including serotonergic and non-serotonergic neurons within the mid-rostrocaudal and caudal DR. Maximal c-Fos protein expression can occur between 1 and 3 h following a stimulus [35] and therefore it is possible that we biased our detection of c-Fos expression toward that associated with recovery from exposure to open-field test, as opposed to that associated with the initial exposure to the stimulus. In total, 4 pairs were tested per day (for a total of 4 days) between 9.00 AM and 1.00 PM. It was considered important to limit the experimental time window because of diurnal variation of c-Fos expression in the DR [32].

### Behavior in the open-field arena

The open-field arena (90 cm width × 90 cm length × 40 cm height) was divided into a 6 × 6 grid of equally-sized squares using black tape. The outer section of the box (OS) was defined as the sum of all squares adjacent to a wall (i.e. 16 out of 36 squares) not including the 4 corner (C) squares. The central region of the box (16 squares) was subdivided into a large (LC) and a small center (SC) of 12 and 4 squares respectively. The test started by placing a rat (LL or HL group only) in the same side of the outer section (halfway along one of the four walls of the box, facing the center) such that the rat could visit the center area first or move to one of the corners. The behavior of each rat in the open-field box was recorded on video and scored afterwards with The Observer^®^ 5.0 software (Noldus Information Technologies BV, Wageningen, The Netherlands, supplied by Tracksys Ltd. Nottingham, UK). For behavior, the data were collapsed for the 4 identical quarters of the box (each containing 3 × 3 squares consisting of 1 C, 4 OS, 3 LC and 1 SC squares each). Time spent in each category of square was recorded. In addition, the fifteen minute open-field test was divided into 3 blocks of 5 minutes and total locomotor activity was scored as the number of square entries in each five minute block. In addition, the frequency of the following behaviors was recorded: stretched-attend posture (stretching forward with the forelimbs extended, often with the back arched in order to maintain a low profile), rearing (standing on hind legs, with our without contact with the sides of the arena), grooming (using paws or tongue to clean/scratch body) and corner-facing (i.e. standing or sitting with the face directed toward the corner of the box). Finally, the latency to visit the large center and any one of the four corners was scored manually.

### Tissue collection and preparation

Following anesthesia, rats were perfused with 0.05 M phosphate buffered saline (PBS: pH 7.4) at 4 °C followed by 4% paraformaldehyde in 0.1 M sodium phosphate buffer (PB: pH 7.4). Brains were removed from the skull and stored in the 4% paraformaldehyde solution at 4 °C. The next day, brains were put in 0.1 M PB for 2 × 12 h, after which they were stored in 30% sucrose in 0.1 M PB until they had sunk. At that point, each brain was cut using a rat brain matrix (RBM-4000C, ASI Instruments, Warren, MI USA) into forebrain and hindbrain sections which were stored at −80 °C until further processing. The hindbrain, including the midbrain raphe complex, was then serially sectioned at 30 μm intervals using a cryostat (Leica CM1900, Leica Microsystems Ltd, Buckinghamshire, UK). Sections were collected as six alternate sets of slices (with each set containing one section at 180 μm intervals throughout the hindbrain) and stored at −20 °C in a cryoprotectant storage buffer (30% ethylene glycol, 20% glycerol in 0.05 M PB; pH 7.4).

### Immunohistochemistry for c-Fos and tryptophan hydroxylase (TrpOH) and cell counting

One set of sections, including the midbrain raphe complex, was used for double immunostaining using primary antisera directed against the protein product of the immediate-early gene *c-fos* (rabbit anti-c-Fos polyclonal antiserum, Cat No. PC38 (Ab-5), 1:10.000; Oncogene Research Products, San Diego, CA, USA), and tryptophan hydroxylase (TrpOH: affinity-purified sheep anti-tryptophan hydroxylase polyclonal antibody, Cat No. 9260-2505, 1:10.000; Biogenesis Ltd, Poole, UK).

**Figure 1:**
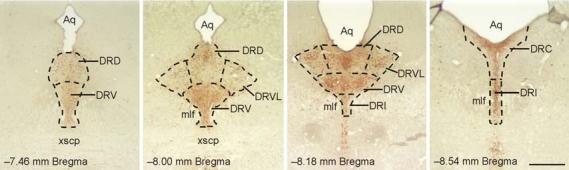
Low magnification photomicrographs illustrating TrpOH/c-Fos-immunostained sections from different rostrocaudal levels of the DR from a control (CO) rat. TrpOH-immunopositive neurons and dendrites can be identified by the brown/orange precipitate within subdivisions of the DR. The subdivisions of the DR analyzed are illustrated by dashed lines (adapted from a standard stereotaxic atlas of the rat brain [42]). Abbreviations: Aq, cerebral aqueduct; DRC, dorsal raphe nucleus, caudal part; DRD, dorsal raphe nucleus, dorsal part; DRI, dorsal raphe nucleus, interfascicular part; DRV, dorsal raphe nucleus, ventral part; DRVL, dorsal raphe nucleus, ventrolateral part; mlf, medial longitudinal fasciculus; xscp, decussation of the superior cerebellar peduncle. Scale bar, 500μm.

Free-floating tissue was incubated at room temperature (RT) in 12-well tissue culture plates and washed in plastic tubs using mesh wells (Corning Costar, Corning, NY, USA), and gently shaken on an orbital shaker throughout double immunostaining. The length of all washes, rinses and pre-incubations was 15 min. Tissue was first washed in 0.05 M PBS, then rinsed in 1% hydrogen peroxide in PBS, washed in 0.05 M PBS, and pre-incubated in PBS containing 0.3% Triton X-100 (PBST); sections were then incubated overnight at RT with rabbit anti-c-Fos antiserum in 0.1% PBST. After 15 h, tissue was washed twice in 0.3% PBST followed by incubation with a biotinylated swine anti-rabbit IgG polyclonal antibody (Cat. No. E0353, 1:200; DakoCytomation Ltd, Cambridgeshire, UK) in 0.1% PBST for 90 min. Tissue was washed twice in 0.3% PBST followed by incubation with Elite ABC reagent (Cat. No. PK-6100, 1:200; Vector Laboratories, Peterborough, UK) in 0.1% PBST for 90 min. Last, tissue was washed in 0.3% PBST, then in PBS, and incubated in SG substrate (Vector Laboratories; Cat. No. SK4700; diluted as recommended by the vendor in PBS) for 23 min. After the chromogen reaction, tissue was immediately washed in PBS, 1% hydrogen peroxide in PBS, PBS, and 0.3% PBST respectively. Sections were then incubated with affinity-purified sheep anti-TrpOH antibody in 0.1% PBST for 18 h. All subsequent steps were identical to those described above for the immunohistochemical localization of c-Fos, except for use of a rabbit anti-sheep secondary antibody (Cat. No. PK-6106, Vector Laboratories) and for the chromogen reaction. For the chromogen reaction, sections were incubated in a solution containing 0.01% diaminobenzidine tetrahydrochloride (DAB) and 0.0015% hydrogen peroxide in PBS for 20 min. Sections were washed twice in PBS to stop the reaction. Brain sections were rinsed briefly in distilled water then mounted on SuperFrost^®^ Microscope slides (Fisher Scientific UK, Leicestershire, UK), dehydrated through an alcohol series and cleared with xylene. Slides were then coverslipped using DPX mounting medium (RA Lamb, London, UK). The color reaction of the c-Fos immunostaining was blue-black and localized to the nucleus while TrpOH immunostaining was orange-brown and localized to the cytoplasm. The numbers of c-Fos-immunopositive serotonergic neurons (i.e., c-Fos-immunopositive/TrpOH-immunopositive cells), the numbers of c-Fos-immunopositive non-serotonergic neurons (i.e., cFos-immunopositive/TrpOH-immunonegative neurons), and the total numbers of TrpOHimmunopositive neurons sampled (i.e. both c-Fos-immunopositive and c-Fos-immunonegative TrpOH-immunopositive neurons) were counted in different regions of the dorsal raphe nucleus at multiple rostrocaudal levels (−7.46, −8.00, −8.18, and −8.54 mm Bregma, Fig. 1) [42]. The subdivisions of the DR studied included the dorsal raphe nucleus, dorsal part (DRD) and dorsal raphe nucleus, ventral part (DRV) at −7.46 mm Bregma, the DRD, DRV and dorsal raphe nucleus, ventrolateral part (DRVL) at −8.00 mm Bregma, the DRD, DRV, DRVL and dorsal raphe nucleus, interfascicular part (DRI) at −8.18 mm Bregma and the dorsal raphe nucleus, caudal part (DRC) and DRI at −8.54 mm Bregma. Although we presume that our estimates of c-Fos+/TrpOH− cells are correct, we cannot assume that both primary antibodies used in this study have identical penetration of the sections; consequently, it should be recognized that estimates of the numbers of c-Fos+/TrpOH− cells are estimates of the numbers of presumptively non-serotonergic cells. Likewise, although we presume that our estimates of c-Fos+/TrpOH+ cells are correct, it is possible that dark immunostaining of serotonergic neurons could hinder our detection of light c-Fos immunostaining; consequently, it should be recognized that estimates of the total numbers of c-Fos+/TrpOH+ neurons may be underestimated. Cell counts were conducted using brightfield microscopy at 400x magnification by an investigator blind to the assignment of treatment groups.

### Data analysis

Data were analyzed using analysis of variance (ANOVA) or, when appropriate, ANOVA with repeated measures analysis. ANOVA analysis was followed, when appropriate, by post hoc analysis using Bonferroni pairwise comparisons using SPSS software (Version 11.5 for Windows, SPSS Inc., Chicago, IL, USA). A Greenhouse-Geisser correction epsilon (ε) was used for repeated measures analysis to correct for potential violation of the sphericity assumption [70]; this correction multiplies both the numerator and the denominator degrees of freedom by epsilon and the significance of the F-ratio is evaluated with the new degrees of freedom, resulting in a more conservative statistical test.

Locomotor activity was analyzed using treatment group (2 levels: LL and HL) as a between-subjects factor and time (3 levels: 0-5 min, 5-10 min, 10-15 min) as a within-subjects factor. The time spent in each square type for the rats exposed to LL or HL conditions was analyzed using independent samples t-tests with a Bonferroni-corrected p value, with an adjusted two-tailed α level for significance of 0.0125. The frequency of each behavior, stretched-attend posture, rearing, grooming and corner facing, was also analyzed using independent samples t-tests.

Cell counts for the numbers of c-Fos-immunopositive serotonergic neurons, the numbers of c-Fos-immunopositive non-serotonergic neurons and the total numbers of TrpOH-immunopositive neurons sampled in the whole DR were analyzed using separate ANOVAs with treatment group (4 levels: CO, HA, LL and HL) as a between-subjects factor. Cell counts for the numbers of c-Fos-immunopositive serotonergic neurons, the numbers of c-Fos-immunopositive non-serotonergic neurons and the total numbers of TrpOH-immunopositive neurons sampled in different subdivisions of the DR were analyzed separately using treatment group as a between-subjects factor and brain region (11 levels) as a within-subjects factor. Significance was accepted for the ANOVAs and post hoc comparisons when *p* < 0.05.

## 3. Results

### Behavior in LL and HL groups

**Figure 2:**
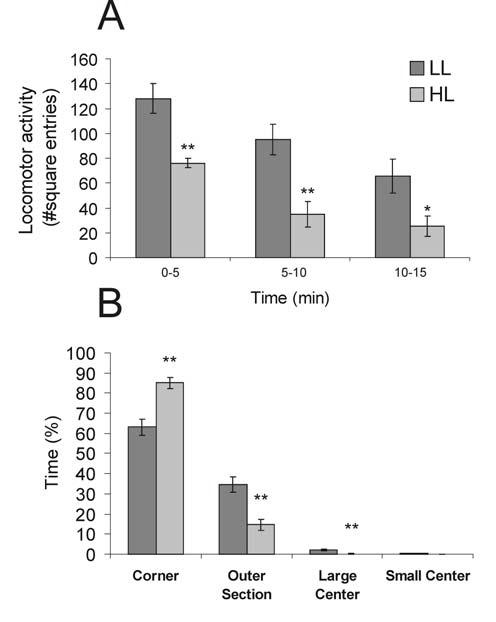
Graphs illustrating the effects of open-field exposure in low-light (LL: 8-13 lux) or high-light (HL: 400-500 lux) conditions on behavior (mean + SEM), including A) locomotor activity, scored as the number of square entries during each five min block of the 15 min open-field test, *p < 0.05, **p < 0.01 versus LL group; post hoc Bonferroni pairwise comparisons, and B) percentage of time spent in each square type, **p < 0.01; Student's *t*-test for independent samples.

Open-field exposure in the LL and HL conditions differentially affected behavior (Figure 2). Rats exposed to the HL condition showed significantly reduced locomotor activity, quantified as the number of square entries, compared to rats exposed to the LL condition (Figure 2A). Factorial ANOVA with repeated measures revealed significant main effects for time block (F(2,28) = 28.25, p < 0.001, ε = 0.887) and light condition (F(1,14) = 18.08, p = 0.001). There was a reduction in motor activity in both the LL and HL rats over time, with HL rats displaying fewer square entries compared with LL rats within each time block. The level of illumination also altered behavioral responses in the open-field based on analysis of the percentage of time spent in each square type (Figure 2B). Rats exposed to the HL condition spent a greater percentage of time in the corners of the box compared to LL rats (85% *versus* 63%; t(14) = −4.37, p < 0.001). Whereas rats exposed to the LL condition spent more time in the outer side (t(14) = 4.22, p < 0.001) and large center (t(14) = 3.406, p = 0.004). There was no difference in the initial behavioral response as measured by the latency to enter a corner of the open-field arena between LL and HL groups (8.6 ± 0.8 and 10.3 ± 2.9 sec respectively; data not shown). The test started halfway along one of the walls of the arena and rats (in both LL and HL conditions) initially moved towards an adjacent corner without passing through the center of the arena. The HL rats took significantly longer to visit the LC (t(14) = -2.38, p < 0.035); 3 of 8 HL rats visited the LC during the 15 minutes compared to 6 of 8 rats in the LL condition. Rats exposed to the HL condition never visited the small center while 5 of 8 rats exposed to the LL condition visited the small center; however, there was no statistical difference between the LL and HL rats in the percentage of time spent in the small center.

**Figure 3:**
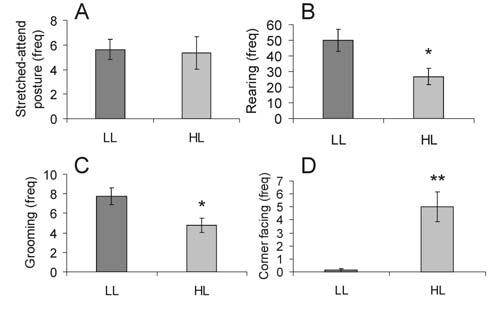
Graphs illustrating the effects of open-field exposure in low-light (LL: 8-13 lux) or high-light (HL: 400-500 lux) conditions on the frequency (mean + SEM) of specific behaviors including A) stretched-attend posture, B) rearing, C) grooming and D) corner facing during the 15 min test. *p < 0.05, **p < 0.01 versus LL group; Student's *t*-test for independent samples.

The frequency of stretched-attend posture was similar in the LL and HL groups (Figure 3A), but rearing (Figure 3B) and grooming (Figure 3C) were significantly reduced in the HL group (t(14) = 2.65, p = 0.019 and t(14) = 2.63, p = 0.020 respectively). In contrast, the frequency of facing the corner was increased in the HL group (Figure 3D; t(14) = −4.27, p = 0.001).

### Analysis of the total numbers of c-Fos-immunopositive serotonergic neurons, c-Fosimmunopositive non-serotonergic cells, and TrpOH-immunopositive neurons in the DR

The total numbers of both c-Fos-immunopositive serotonergic neurons (double-immunostained; F(3,28) = 5.612, p = 0.004) and c-Fos-immunopositive non-serotonergic cells (F(3,28) = 144.40, p, 0.001) within the DR were significantly higher in rats exposed to either the LL or the HL condition in the open-field test, compared to both CO and HA rats (Table 1). Likewise, the percentage of serotonergic neurons that were c-Fos-immunopositive was also significantly higher in rats exposed to either the LL or the HL condition compared to CO and HA rats (Table 1). There were no differences among the treatment groups in the total numbers of TrpOH-immunopositive neurons sampled within the DR (F(3,28) = 1.58, p = 0.216; Table 1).

### c-Fos-immunopositive serotonergic neurons in subdivisions of the DR

**Figure 4:**
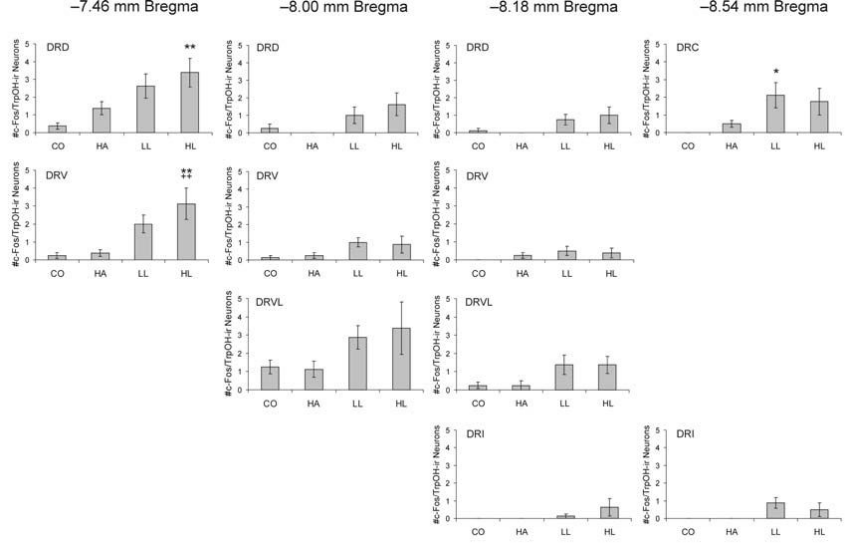
Graphs illustrating the effects of handling, low-light, or high-light open-field exposure, compared to home cage controls, on the number of c-Fos-immunopositive/TrpOH-immunopositive cells (mean + SEM) within different subdivisions of the dorsal raphe nucleus at the four rostrocaudal levels analyzed. Rats were left undisturbed in their home cages (CO), briefly handled (HA), or exposed to low-light (LL: 8-13 lux) or high-light (HL: 400-500 lux) conditions in an open-field arena for 15 min. *P < 0.05, **P< 0.01 versus CO group; ++P < 0.01 versus HA group; post hoc Bonferroni pairwise comparisons. For abbreviations, see Fig. 1 legend.

Factorial repeated measures ANOVA of the numbers of c-Fos-immunopositive/TrpOH-immunopositive (serotonergic) neurons within specific subdivisions of the DR revealed significant main effects for both DR subdivision (Figure 4; F(10,280) = 14.44, P < 0.001, ε = 0.44) and treatment group (F(3,28) = 5.69, P < 0.005). The interaction approached statistical significance (F(30,280) = 1.77, P = 0.054, ε = 0.44). Post hoc analysis showed no significant differences in the numbers of c-Fos-immunopositive/TrpOH-immunopositive cells between the CO and HA rats in any brain region examined. There were small but statistically significant increases in the number of double-immunostained cells in the HL, but not LL condition, compared to CO rats in the DRD (−7.46 mm Bregma) and compared to CO and HA rats in the DRV (−7.46 mm Bregma). There was also a significant increase in the LL, but not HL condition, compared to CO and HA rats in the DRC (−8.54 mm Bregma). There were no significant differences between LL and HL rats in any of the brain regions examined.

### c-Fos-immunopositive non-serotonergic cells in subdivisions of the DR

**Figure 5:**
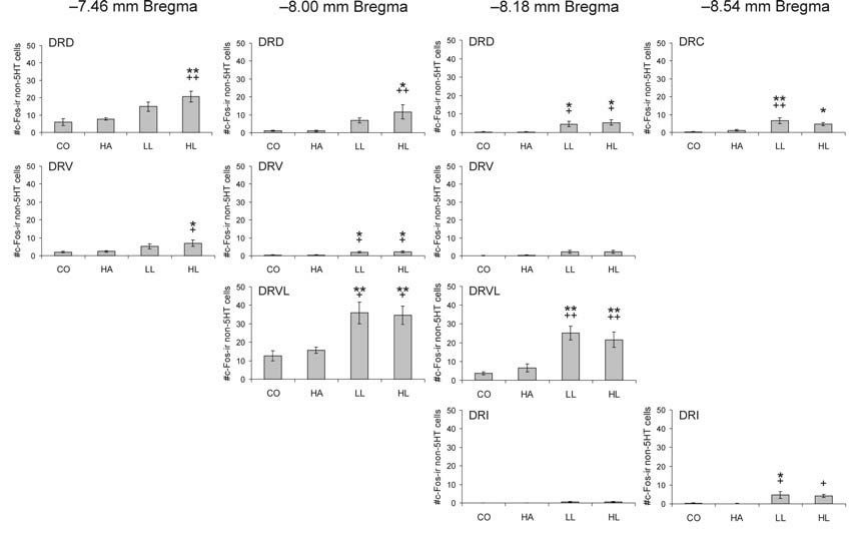
Graphs illustrating the effects of handling, low-light, or high-light open-field exposure, compared to home cage controls, on the number of c-Fos-immunopositive/TrpOH-immunonegative cells (mean + SEM) within different subdivisions of the dorsal raphe nucleus at the four rostrocaudal levels analyzed. Rats were left undisturbed in their home cage (CO), briefly handled (HA) or exposed to low-light (LL: 8-13 lux) or high-light (HL: 400-500 lux) conditions in an open-field arena for 15 min. *P < 0.05, **P< 0.01 versus CO group. +P < 0.05, ++P < 0.01 versus HA group; post hoc Bonferroni comparisons. For abbreviations, see Fig. 1 legend.

**Figure 6:**
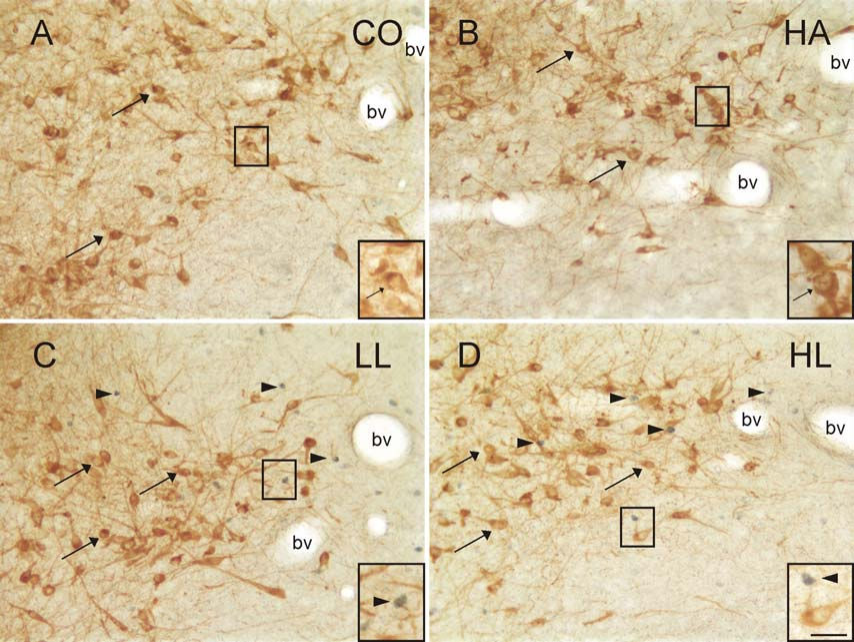
Photomicrographs illustrating c-Fos-immunopositive nuclei and TrpOH-immunopositive neurons in the mid-rostrocaudal DRVL (−8.18 mm Bregma) of rats exposed to A) CO, B) HA, C) LL, and D) HL conditions. Black boxes indicate regions shown at higher magnification in insets in the lower right hand corner of each panel. Arrowheads indicate examples of c-Fos-immunopositive cells (blue/black nuclear staining); arrows indicate TrpOH-immunopositive (serotonergic) neurons (brown/orange cytoplasmic staining). c-Fos-immunopositive/TrpOH-immunopositive neurons were rarely observed. Abbreviation: bv, blood vessels characteristic of the DRVL region at this rostrocaudal level. Scale bar, 50 μm, inset 25 μm.

Factorial repeated measures ANOVA of the numbers of c-Fos-immunopositive/TrpOH-immunonegative (non-serotonergic) cells within specific subdivisions of the DR revealed a significant interaction between treatment group and subdivision (Figure 5; F(30,280) = 5.81, P < 0.001, ε = 0.32). The main effects for treatment group (F(3,28) = 16.84, p < 0.001) and subdivision (F(10,280) = 78.47, p < 0.001, ε = 0.32) were also significant. As noted with the double-immunostained cells, post hoc analysis revealed no significant differences between CO and HA rats in any of the regions examined. Open-field exposure in both the LL and the HL conditions significantly increased c-Fos immunoreactivity in non-serotonergic cells compared to CO and HA rats widely throughout the DR, including the DRV and DRVL at −8.00 mm Bregma, and the DRD and DRVL at −8.18 mm Bregma. In rats exposed to the open-field, c-Fos-immunopositive non-serotonergic cells were found interspersed among the c-Fos-immunonegative serotonergic neurons in the DRVL, particularly in the lateral part of the DRVL (Fig. 6). There were significant increases in the HL, but not LL condition, compared to CO and HA rats in the DRD and DRV at −7.46 mm Bregma and the DRD at −8.00 mm Bregma and in the LL, but not the HL, condition compared to CO and HA rats in the DRC and DRI (−8.54 mm Bregma). There were also significant increases in the HL group compared to the CO group in the DRC (−8.54 mm Bregma) and compared to the HA group in the DRI (−8.54 mm Bregma). There were no significant differences between the LL and HL groups in any of the brain regions examined.

### TrpOH-immunopositive neurons in subdivisions of the DR

The number of TrpOH-immunopositive cells sampled within each subdivision of the DR varied across subdivisions (Table 2: F(10,280) = 188.72, P < 0.001, ε = 0.60). However, the total number of TrpOH-immunopositive cells sampled within each subdivision was similar across treatment groups and there was no interaction between subdivision and treatment group.

## 4. Discussion

Exposure of rats to the HL condition in an open-field increased anxiety-related behavior relative to rats exposed to LL conditions. Exposure of rats to an open-field arena, with either low or high levels of illumination, increased the expression of c-Fos in serotonergic and non-serotonergic neurons within subdivisions of the dorsal raphe nucleus, relative to home cage control rats. Exposure to the open-field arena in the HL condition resulted in small but significant increases in c-Fos expression in serotonergic neurons within the rostral part of the dorsal raphe nucleus (DR). In contrast, with the exception of a small but significant increase in c-Fos expression in serotonergic neurons within the DRC, exposure to the open-field arena had no effect on c-Fos expression in serotonergic neurons within the mid-rostrocaudal and caudal parts of the DR, suggesting that this stimulus was below the threshold required to induce an intense and prolonged activation of serotonergic neurons in this region. Furthermore, despite the observation that exposure of rats to a HL condition, relative to a LL condition, increased anxiety-related behavioral responses, exposure to the HL condition had no effect on the number of c-Fos-immunopositive serotonergic or non-serotonergic neurons relative to exposure to the LL condition. It is possible that there were increases in the neuronal activity of serotonergic and non-serotonergic neurons in the DR in HL rats compared to LL rats, but that the increases in neuronal activity were below the threshold required to induce the expression of c-Fos protein.

### Exposure to the HL condition increased anxiety-related behavior compared to the LL group

Exposure of rats to the HL condition, relative to the LL condition, increased multiple measures of anxiety-related behavior. Increasing the intensity of illumination of the open-field arena reduced locomotor activity and increased avoidance of the center of the arena. In addition, rearing and grooming were reduced under the HL condition while the duration of time spent in the corners of the apparatus and the frequency of a stereotypical behavior of facing the corners of the apparatus were increased. Facing the corner is interpreted as a coping style to avoid the exposure to the bright light and the open surface of the arena. It is important here to highlight the differences in distributions of percent time spent in specific regions of the arena and the number of visits to specific regions of the arena. The combination of these measures elucidated that rats had a strong preference to stay in the corner of the open-field arena, while they moved quickly from one corner to the next. Activity in the center of the arena was low in both groups, but significantly reduced in the HL rats, none of which visited the small center, compared with 5/8 rats in the LL group that did visit the small center. Previous studies have demonstrated that the initial response to the open-field arena is behavioral activation and exploration, although subtle procedural distinctions can influence the response [52]. Our data confirm previous findings that behavioral activity in the open-field arena, studied here in both LL and HL rats, decreases over time (e.g.[12;41;72]). Overall, the behavioral activation in rats exposed to HL conditions was lower than the behavioral activation in rats exposed to LL conditions. Altogether, these data show that exposure to the HL condition increased anxiety-related behavior in the open-field arena, consistent with previous findings in a circular open-field test in male and female Wistar rats [41]. Consequently, we would expect to find differences in c-Fos expression in subsets of serotonergic neurons in rats exposed to the LL and HL conditions if subsets of serotonergic neurons contribute to the differential expression of these anxiety-related behavioral responses.

### Exposure to the HL condition in the open-field increase c-Fos expression in serotonergic neurons in the rostral DR

Exposure of rats to an open-field arena in high-light conditions resulted in a small but significant increase in c-Fos expression in serotonergic neurons within the rostral DR, including its dorsal and ventral parts. This region of the DR gives rise to serotonergic projections to forebrain systems involved in somatic motor responses including the substantia nigra and caudate putamen [28;29;65] and includes an anatomically distinct mesostriatal serotonergic system [37]. In support of a functional association between serotonergic neurons within the rostral DR and voluntary motor responses, studies by Greenwood and colleagues have demonstrated that rats that are allowed 6 weeks of voluntary freewheel running respond with increases in 5-HT_1A_ autoreceptor mRNA expression and decreases in 5-HT_1B_ receptor mRNA that are restricted to the rostral to mid–rostrocaudal regions of the DR [18;19].

The finding that exposure of rats to the open-field arena in high-light conditions increased c-Fos expression in serotonergic neurons in the rostral DR, but not in other regions of the DR is consistent with previous studies demonstrating a dissociation of the activation of mesostriatal serotonergic systems (which are located primarily in the rostral DR) and mesolimbocortical serotonergic systems (which are located primarily in the caudal DR and median raphe nucleus). Studies by Daugherty and colleagues [8] have demonstrated that treatment of rats with morphine increases extracellular serotonin concentrations in the caudate putamen within the dorsal striatum but not in forebrain targets of mesolimbocortical serotonergic systems including the hippocampus. In contrast, exposure of rats to sound stress increases extracellular serotonin concentrations in the hippocampus, but not in the caudate nucleus. These studies suggest that there is a dissociation between the activation of mesostriatal and mesolimbocortical serotonergic systems under different experimental conditions.

The effect of exposure to the open-field arena on c-Fos expression in the rostral DR under HL conditions in the present study was small and, because rats exposed to the HL condition responded with less locomotor activity than rats exposed to LL conditions, not directly associated with locomotor activity. Studies in behaving animals have demonstrated that the firing rates of DR serotonergic neurons are correlated with muscle tone [30] and it is possible that the small but significant effects of exposure to the open-field under HL but not LL conditions in the rostral DR were associated with a greater muscle tone in rats exposed to the HL condition. Thus, despite the anatomical and functional studies outlined above, it remains possible that increases in c-Fos expression within serotonergic neurons within the rostral DRD and DRV following exposure to the HL condition may be associated with anxiety-related behavior or its physiological correlates, while increases in c-Fos expression within serotonergic neurons within the DRC may be associated with locomotion, or its physiological correlates.

Although it is difficult to identify the physiological or behavioral correlates of the increase in c-Fos expression in serotonergic neurons within the rostral DR in rats exposed to the HL condition, compared to either CO or HA rats, this effect is consistent with previous studies demonstrating an association between the firing rate of DR serotonergic neurons and behavioral state. The majority of serotonergic neurons in behaving cats [47;55], and rats [71], referred to as Type I serotonergic neurons, have neuronal firing rates that are highly correlated with behavioral arousal. In both cats and rats, however, other topographically organized subsets of serotonergic neurons have been identified that have neuronal firing rates that are not highly correlated with behavioral state [47;55]. Of interest is the finding that of the cat serotonergic neurons that display higher neuronal firing rates during active waking, relative to quiet waking (Type IA, IB, and IIA), the vast majority of these (Type IA and IB serotonergic neurons) are located in the rostral DR, near the oculomotor nucleus [55]. These data are consistent with the present study and together these studies support a close association between behavioral arousal or vigilance and the activity of serotonergic neurons within the rostral DR.

### Exposure to the open-field in either the LL or HL condition did not affect c-Fos expression within the mid-rostrocaudal and caudal DR

Of particular interest in this study is the finding that exposure to the open-field arena, either in the LL condition or the HL condition, had little or no effect on c-Fos expression in serotonergic neurons within the mid-rostrocaudal and caudal DR compared to the CO or HA groups. This is in contrast to previous studies which have described effects of multiple anxiogenic drugs [1], anxiety-related neuropeptides [64], or anxiety-related stimuli with strong elements of aversiveness [13;16] on c-Fos expression in serotonergic neurons in these regions. Furthermore, data from the present study demonstrate that an increase in anxiety-related behavior (i.e. as observed in the HL relative to the LL condition) is not necessarily associated with an increase in c-Fos expression in serotonergic neurons within the mid-rostrocaudal and caudal parts of the DR. These data suggest that either the intensity of the anxiogenic stimulus in the present study was below the threshold for widespread activation of serotonergic neurons within these regions of the DR, or that the activation of serotonergic neurons within these regions by multiple anxiogenic drugs or anxiogenic stimuli, as described in previous studies, is unrelated to the quantitative aspects of the anxiety-related behavioral responses.

As serotonergic systems within the DR, particularly those within the mid-rostrocaudal and caudal DR, are thought to play a role in facilitation of anxiety-related behavior, and as rats exposed to the HL condition in the open-field test responded with increased anxiety-related behavior, we expected to find greater increases in c-Fos expression in serotonergic neurons in rats exposed to the HL condition, compared to the LL condition in the open-field. However, we found no evidence for greater c-Fos expression in serotonergic or non-serotonergic neurons in rats exposed to the HL condition, compared to rats exposed to the LL condition, within any subdivision of the DR studied.

Based on several lines of evidence, it remains possible that serotonergic systems arising from the DR are necessary for the HL-induced facilitation of anxiety-related behavior in open-field test. Several studies suggest that disruption of the normal serotonergic tone within the DR can have anxiolytic effects when rats are tested under aversive conditions. For example, intra-DR injections of the 5-HT_1A_ receptor agonists (±)-8-hydroxy-dipropylaminotetraline (8-OH-DPAT) and 5-carboxamidotryptamine (5-CT), the 5-HT_1A_ receptor partial agonists buspirone and ipsapirone, or the GABA_A_ receptor agonist muscimol, have anxiolytic effects in the social interaction test when tested under high-light, unfamiliar conditions [24;27;44]. In other studies, intra-DR injections of 8-OH-DPAT, buspirone, and gepirone increased social interaction in the social interaction test (an anxiolytic effect) when rats were tested under high-light, unfamiliar conditions, but not when they were tested under low-light, familiar conditions [24], suggesting that a different level of activity of DR neurons under these two conditions contributes to the different behavioral responses. Likewise, intra-DR 8-OH-DPAT injections have anxiolytic effects in other tests with aversive or highly aversive conditions, including the elevated plus-maze test [11], Vogel conflict test [23], shock-induced ultrasonic vocalization test, and Geller-Seifter conflict test [57]. The anxiolytic effects of intra-DR injections of 8-OH-DPAT are prevented by administration of the 5-HT_1A_ receptor antagonists tertatolol [27] and WAY-100635 [N-[2-[4-(2-methoxyphenyl)-1-piperazinyl]ethyl]-N-(2-pyridinyl) cyclo-hexane-carboxamide trihydrochloride] [48], providing further evidence for a specific role for 5-HT_1A_ receptors. The anxiolytic effects of intra-DR injections of 8-OH-DPAT in these tests may be mediated by activation of 5-HT_1A_ somatodendritic autoreceptors on serotonergic neurons, which is known to inhibit serotonergic neuronal firing rates and to decrease serotonergic neurotransmission in projection areas [26;58;63]. Consistent with this hypothesis, selective lesion of serotonergic systems using i.c.v. treatment with 5,7-dihydroxytryptamine or i.p. treatment with parachlorophenylalanine are anxiolytic in the highly aversive shock-induced ultrasonic vocalizaton test [57]. However, 5-HT_1A_ receptors are also located on non-serotonergic neurons in the DR [34] and could contribute to regulation of anxiety-related behavioral responses. Taken together, these data point toward an important role for activation of DR serotonergic systems in the facilitation of anxiety-related behavior tested under aversive conditions, such as a high light, unfamiliar environment. It is possible that in the present study serotonergic neurons in the DR were engaged at a higher level of activity in the HL condition, compared to the LL condition, but the higher level of activity was below the threshold required for induction of c-Fos protein.

Rapid and intense activation of stress coping mechanisms may explain why we did not detect widespread increases in c-Fos expression in rats exposed to the HL condition compared to those subjected to the LL condition. For example, stress coping mechanisms may be engaged that reduce the activation of serotonergic system to a period of time which is insufficient to induce c-Fos expression. Indeed studies using microdialysis to measure serotonin release in Fisher 344 and Sprague-Dawley rats exposed to the elevated plus-maze have found that increases in extracellular serotonin concentrations in the ventral hippocampus are very brief (≤ 20 min) [49;50]. It should be noted that male Wistar rats did not show the elevated plus-maze-induced increase in serotonin release [3;49].

### Exposure of rats to the open-field in LL and HL conditions increased c-Fos expression in non-serotonergic cells

Rats exposed to the open-field, relative to home cage control rats, had increased c-Fos expression in non-serotonergic neurons throughout the DR, independent of the level of illumination. Indeed, the majority (>98%) of c-Fos-positive cells in rats exposed to the open-field arena in the present study were non-serotonergic. The DRVL contained the largest numbers of c-Fos-immunopositive non-serotonergic cells in rats exposed to LL or HL conditions in the open-field arena. This finding is consistent with a previous study demonstrating that exposure of mice to the elevated plus-maze increased c-Fos expression in the DRVL region measured 2 h after maze exposure (although the previous study did not determine if the c-Fos expression was in serotonergic or non-serotonergic neurons) [60]. It is possible that activation of inhibitory local GABAergic interneurons, which are known to be prevalent in the DRVL region [9;33] contributed to the low levels of c-Fos expression within DR serotonergic neurons in the present study. It has recently been reported that exposure to the forced swim test also increases c-Fos expression in the DRVL region [51]. Double immunostaining revealed that 85% of the c-Fos-immunopositive cells in this region were GABAergic while only 4% were serotonergic. The authors hypothesized that activation of GABAergic neurons in the DRVL might be responsible for the decreases in serotonergic neurotransmission that have been described in rats following exposure to swim stress. However, this region contains both local GABAergic and glutamatergic interneurons that are known to influence the activity of DR serotonergic neurons [33]. Furthermore, the DR contains diverse populations of neurons expressing many different neurotransmitters and neuropeptides [30;40] and identification of the neurochemical phenotypes of these DR non-serotonergic neurons affected by exposure to LL or HL conditions in the open-field will require further study.

### Summary

Overall, the increased c-Fos expression observed in subpopulations of serotonergic and non-serotonergic neurons in the DR may be associated with the facilitation of anxiety-related behavior. However, there are other possibilities that should be considered. As mentioned above, the increased c-Fos expression may be associated with aspects of behavioral arousal, vigilance, or muscle tone, but could also be associated with changes in physical parameters such as cutaneous or core body temperature, or even adaptive or coping mechanisms following the return of rats to their home cages, particularly given the 2 h interval before anesthesia. For example, the large increases in c-Fos expression in non-serotonergic neurons within the DRVL regions could reflect activation of GABAergic interneurons, limiting the intensity or duration of activation of serotonergic systems, when it is perceived by the rat that the stimulus is controllable or escapable. It is important to recognize the range of possible associations that may exist between the behavioral test and c-Fos expression within the DR.

### Conclusions

This study demonstrates for the first time that behavioral arousal associated with exposure to a novel environment is associated with small but significant increases in c-Fos expression within a subset of serotonergic neurons located within the rostral DR, a region that gives rise to mesostriatal serotonergic projections and contains a high density of serotonergic neurons that display increases in neuronal firing rates during active waking states versus quiet waking states in rats. Although the level of illumination in the open-field arena affected anxiety-related behavioral responses, data from this study suggest exposure to the HL condition did not result in an intense and prolonged activation of DR serotonergic neurons. These findings do not exclude, however, the possibility that exposure to the HL condition increased serotonergic neuronal activity but that the effect was below the threshold for induction of c-Fos expression. Together, these findings support an anatomical and functional topographical organization of the DR and raise interesting questions related to the functional relationship between DR serotonergic systems and anxiety-related behavior.

## Figures and Tables

**Table 1:** Numbers of cells counted across all subdivisions of the dorsal raphe nucleus (mean ± SEM)

Counts	Area	CO	HA	LL	HL	Group Effect
c-Fos+/TrpOH+	All levels	2.6 ± 0.4	4.1 ± 1.0	15.3 ± 2.5^A^^,^^B^	18.0 ± 5.9^A^^,^^B^	P < 0.005

c-Fos+/TrpOH−	All levels	27 ± 5	36 ± 3	109 ± 16 A^,^^B^	115 ± 15 A^,^^B^	P < 0.001
Total TrpOH+	All levels	1241 ± 36	1202 ± 33	1187 ± 32	1149 ± 29	P = 0.28
						

%Double/total TrpOH	All levels	0.20 ± 0.03%	0.35 ± 0.10%	1.32 ± 0.23%^A^^,^^B^	1.60 ± 0.39%^A^^,^^B^	P < 0.005
%Double/total c-Fos	All levels	10.4 ± 2.1%	10.7 ± 2.2%	11.8 ± 0.8%	12.1 ± 1.7%	P = 0.89

A P < 0.05 versus CO rats

B P < 0.05 versus HA rats.

Abbreviations: c-Fos+/TrpOH+, c-Fos-immunopositive/TrpOH-immunopositive neurons; c-Fos+/TrpOH−, c-Fos-immunopositive/TrpOH-immunonegative cells; %Double/totalTrpOH, the percentage of c-Fos-immunopositive/TrpOH-immunopositive neurons/the total number of TrpOH-immunopositive neurons; %Double/total c-Fos, the percentage of c-Fos-immunopositive/TrpOH-immunopositive neurons/the total number of c-Fos-immunopositive cells

**Table 2:** Numbers of TrpOH-immunopositive cells sampled in subdivisions of the dorsal raphe nucleus (mean ± SEM)

Rostrocaudal Level	Area	CO	HA	LL	HL	Group Effect
−7.46 mm	DRD	137 ± 6	138 ± 9	138 ± 9	125 ± 9	P = 0.62
−8.00 mm	DRD	96 ± 7	98 ± 5	104 ± 9	102 ± 6	P = 0.86
−8.18 mm	DRD	103 ± 7	100 ± 5	92 ± 4	96 ± 9	P = 0.67

−7.46 mm	DRV	146 ± 10	127 ± 7	128 ± 9	133 ± 10	P = 0.43
−8.00 mm	DRV	208 ± 5	220 ± 13	216 ± 14	203 ± 7	P = 0.66
−8.18 mm	DRV	105 ± 12	98 ± 8	99 ± 10	85 ± 7	P = 0.52

−8.00 mm	DRVL	172 ± 5	164 ± 12	149 ± 7	156 ± 13	P = 0.40
−8.18 mm	DRVL	103 ± 6	88 ± 4	87 ± 8	93 ± 9	P = 0.33

−8.18 mm	DRI	28 ± 3	27 ± 2	24 ± 2	26 ± 3	P = 0.64
−8.54 mm	DRI	50 ± 5	51 ± 4	56 ± 6	46 ± 4	P = 0.53

−8.54 mm	DRC	92 ± 6	93 ± 7	94 ± 3	86 ± 4	P = 0.69
